# Supplementary material for: Why Is the Grass the Best Surface to Prevent Lameness? Integrative Analysis of Functional Ranges as a Key for Dairy Cows’ Welfare
Source: Animals (Basel). 2022 Feb 17;12(4):496. doi: 10.3390/ani12040496 (PMC8868409; doi:10.3390/ani12040496)
Supplement: Supplementary file 1 [file animals-12-00496-s001.zip › animals-1549430 - SI.pdf]

animals

MDPI

Supplementary material

Why Is the Grass the Best Surface to Prevent Lameness? Integrative Analysis of Functional Ranges as a Key for Dairy Cows' Welfare

Paul Medina-González [1,2,*], Karen Moreno [3,*] and Marcelo Gómez [4]

[1] Departamento de Kinesiología, Facultad de Ciencias de la Salud, Universidad Católica del Maule, Talca 3480112, Chile
[2] Programa de Doctorado en Ciencias Veterinarias, Universidad Austral de Chile, Valdivia 5110566, Chile
[3] Laboratorio de Paleontología, Facultad de Ciencias, Instituto de Ciencias de la Tierra, Universidad Austral de Chile, Valdivia 5110566, Chile
[4] Instituto de Farmacología y Morfofisiología, Facultad de Ciencias Veterinarias, Universidad Austral de Chile, Valdivia 5110566, Chile; marcelogomez@uach.cl
* Correspondence: paulmedinagonzalez@gmail.com or pmedina@ucm.cl (P.M.-G.); karenmoreno@uach.cl (K.M.); Tel.: +56-71-2413622 (P.M.-G.)

Citation: Medina-González, P.; Moreno, K.; Gómez, M. Why Is the Grass the Best Surface to Prevent Lameness? Integrative Analysis of Functional Ranges as a Key for Dairy Cows' Welfare. *Animals* **2022**, *12*, 496. https://doi.org/10.3390/ani12040496

Academic Editor: Miriam Annika Vogt

Received: 25 December 2021
Accepted: 8 February 2022
Published: 17 February 2022

Publisher's Note: MDPI stays neutral with regard to jurisdictional claims in published maps and institutional affiliations.

Copyright: © 2022 by the authors. Licensee MDPI, Basel, Switzerland. This article is an open access article distributed under the terms and conditions of the Creative Commons Attribution (CC BY) license (https://creativecommons.org/licenses/by/4.0/).

Table S1. Documented prevalence of dairy cow lameness according to floor type.

Reference	Country [Sample characteristics]	Prevalence				
		Grass	Sand	Rubber	Asphalt	Concrete
Solano et al., 2015 [22]	Canada [5637 cows in 141 herds]			19%		64%
Frankena et al., 2009 [29]	Netherlands [240 cows in 12 herds]	1%				40.6%
Fabian et al., 2014 [31]	New Zealand [23949 cows in 59 herds]	8.3%				
Husfeldt & Endres, 2012 [33]	United States [37271 cows in 34 herds]		14.4%		19.8%	
Rahman et al., 2014 [35]	Bangladesh [2103 cows in 87 farms]			18.3%	13.3%	68.4%
Salfer et al., 2018 [36]	United States [12 herds]	22.5%	21.5%		40.9%	
Faye & Lescouret, 1989 [58]	France [80 farms]	2.9%				19.8%
Frankena et al., 1992[59]	Netherlands [1141 dairy calves]	4.6%				44.6%
Cook et al., 2003[68]	United States [3621 cows in 30 herds]		11.4%	17.4%		
Vokey et al., 2001 [69]	United States [120 primi-multiparous]		5%	5%		32%
Espejo et al, 2006[70]	United States [5626 cows in 53 herds]		17.1%	27.9%		
Cramer, 2007 [74]	Canada [13530 cows in 204 herds]			23.7%		52.6%
Cook et al., 2016 [75]	United States [9690 cows in 66 herds]		11%	17.5%		

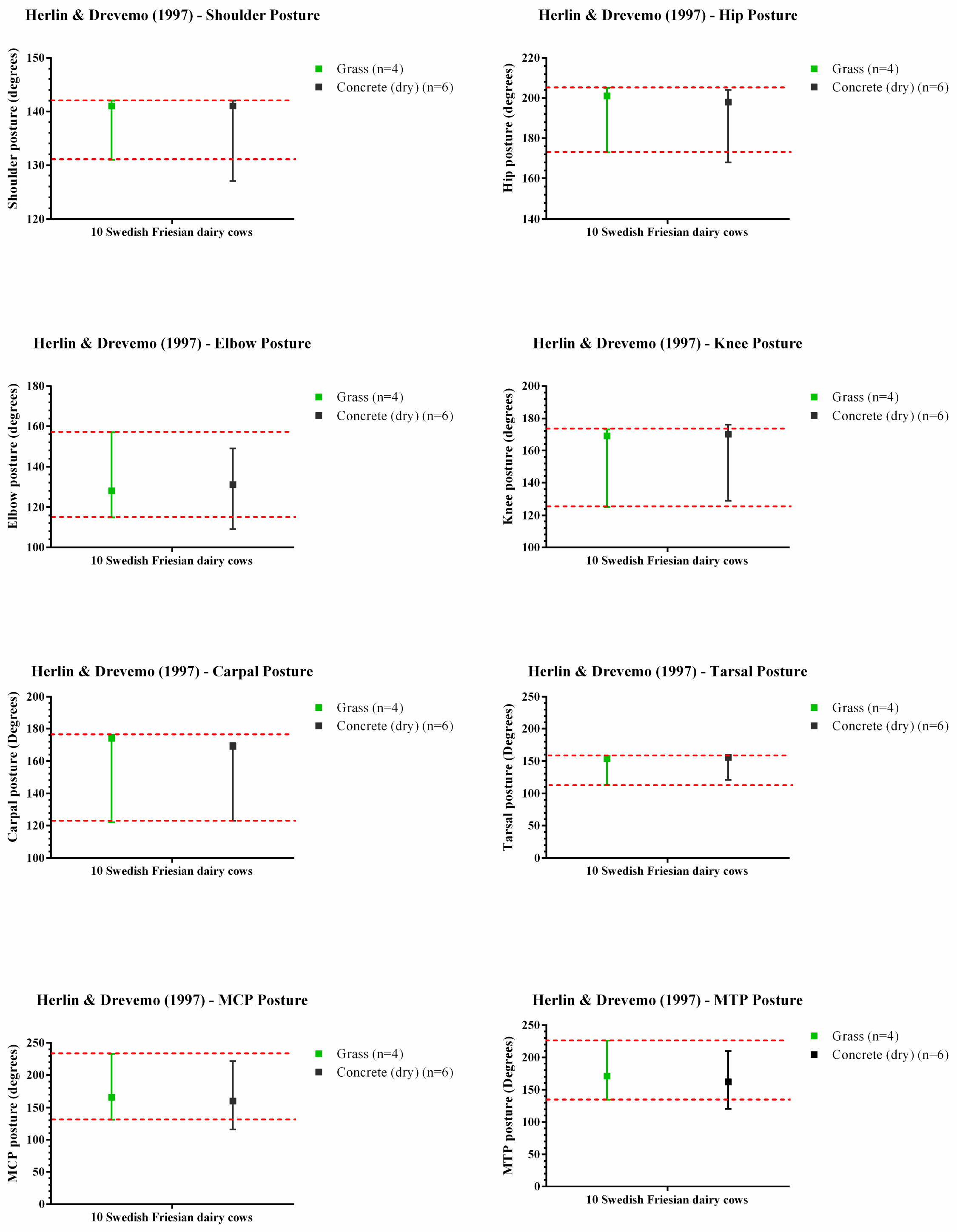

Figure S1. Posture parameters for grass and concrete surfaces.

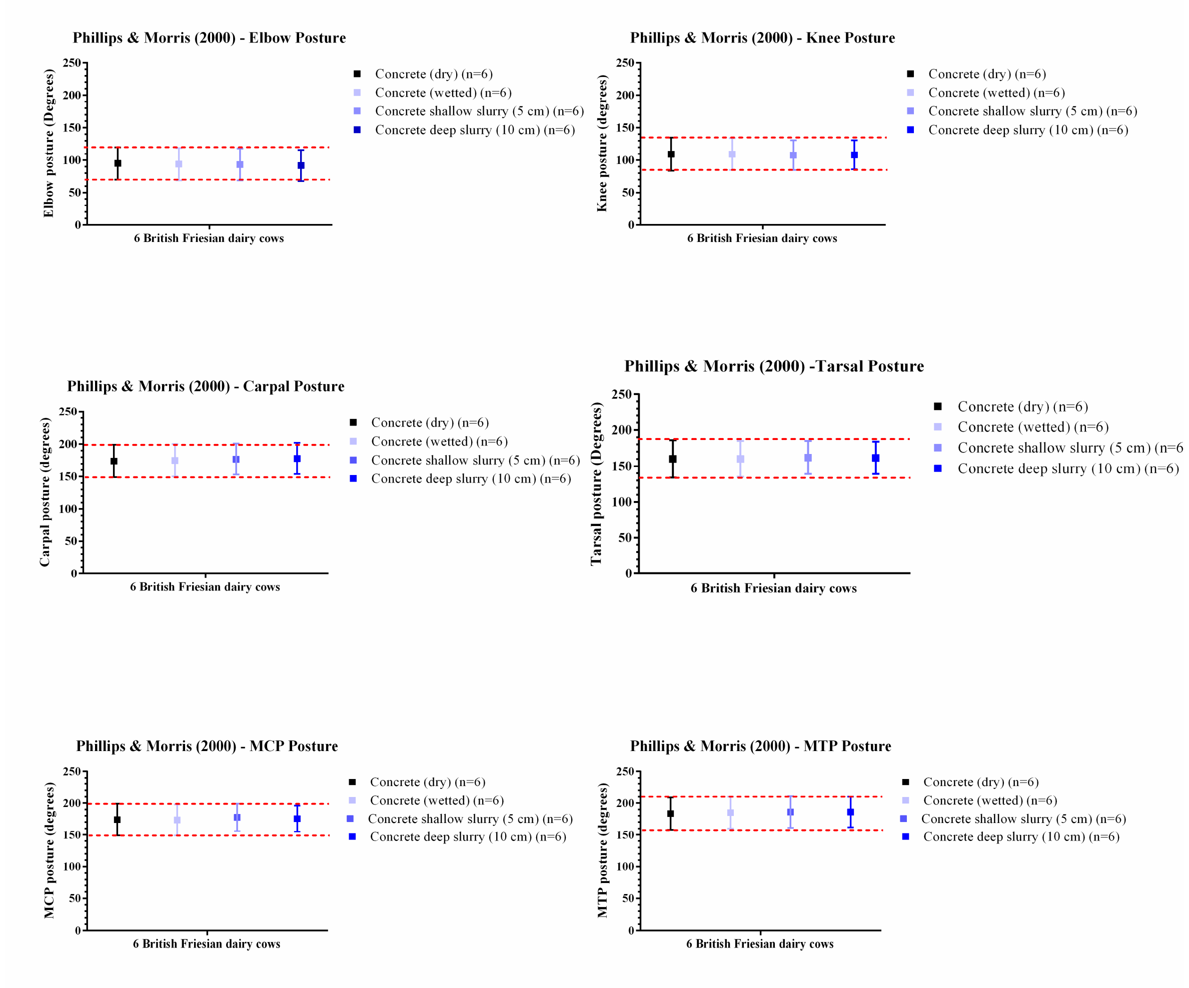

Figure S2. Posture parameters for concrete surface (soak factor).

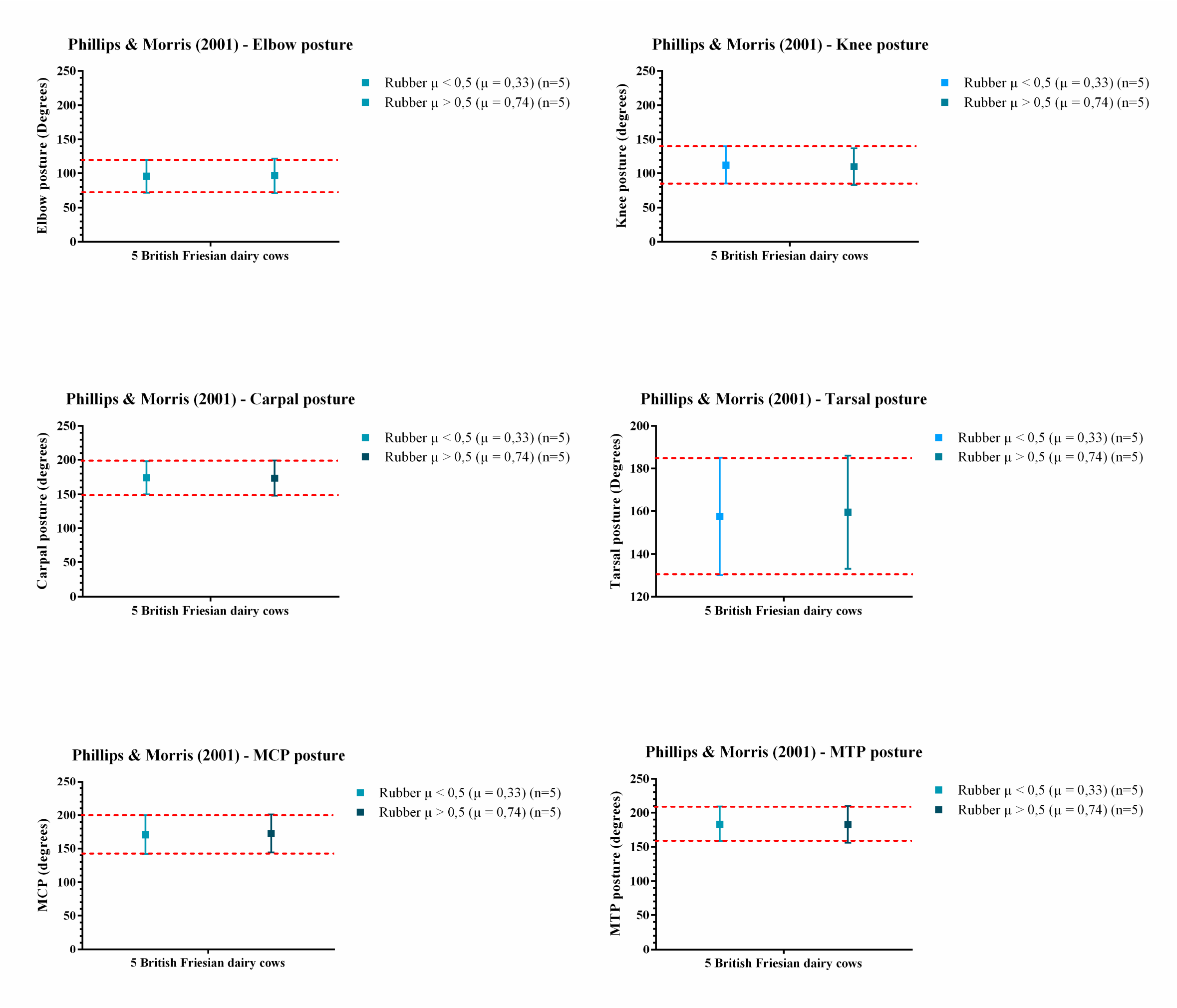

Figure S3. Posture parameters for rubber surface (friction factor).

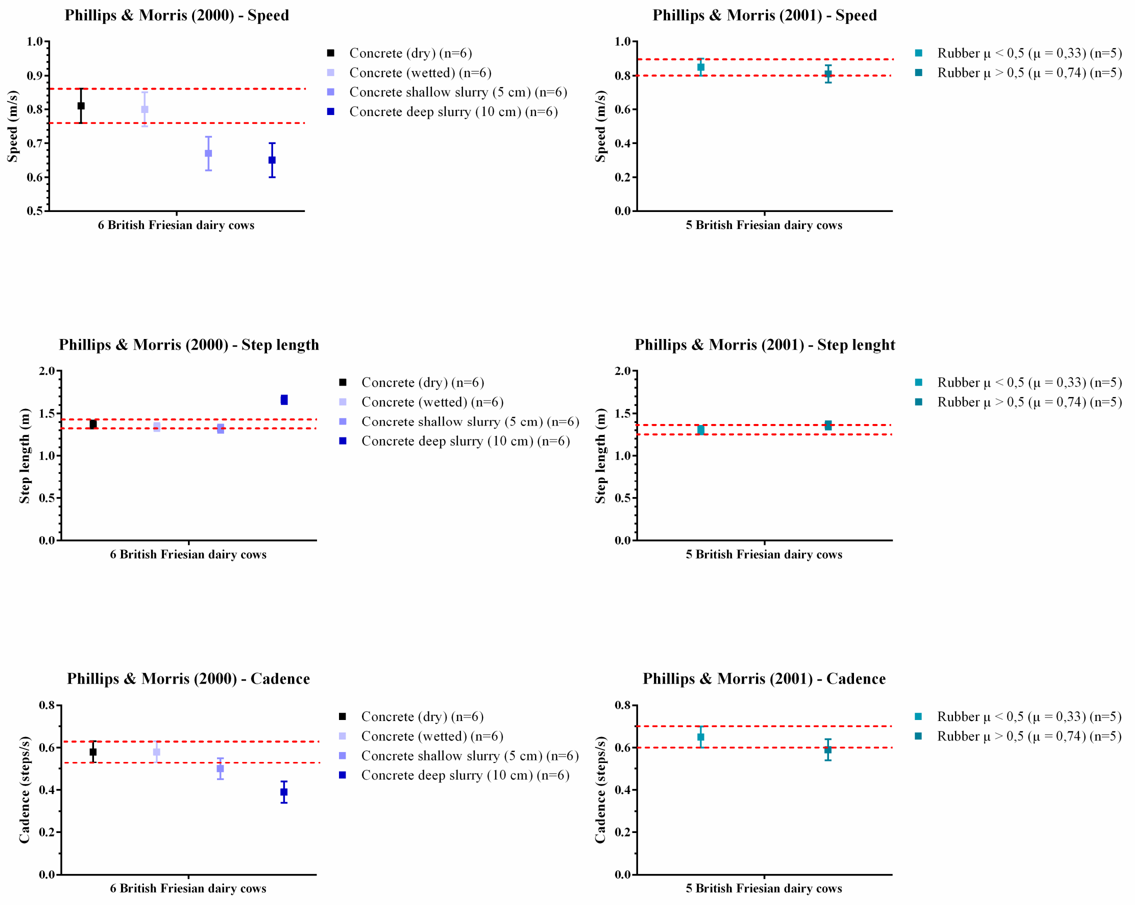

Figure S4. Kinematics parameters for rubber (friction factor) and concrete (soak factor) surfaces.

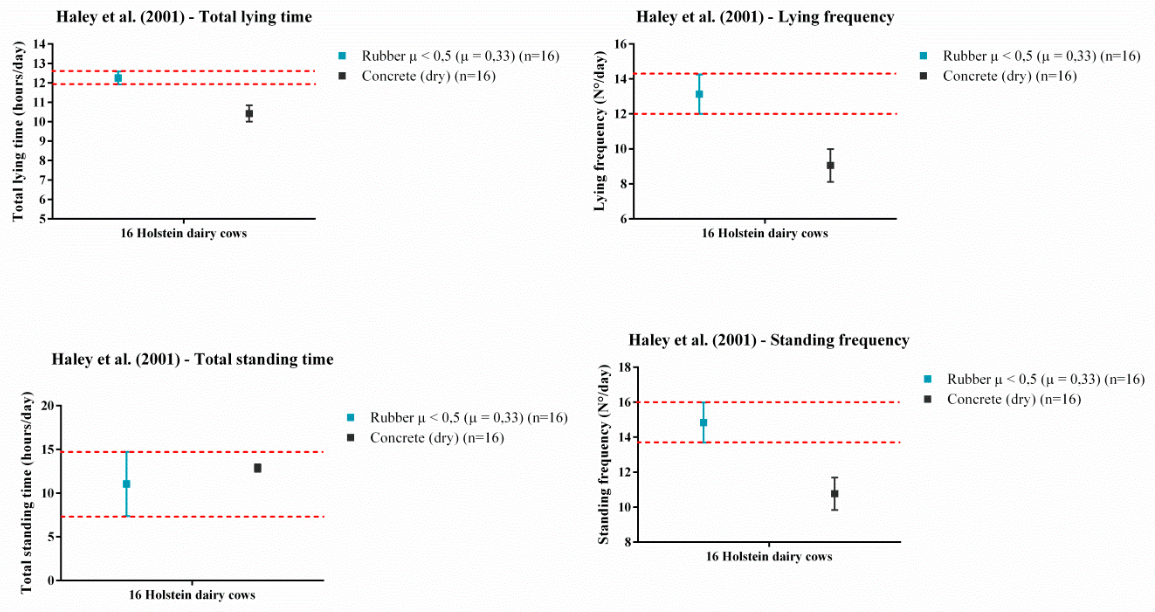

Figure S5. Behavior parameters for rubber and concrete surfaces.

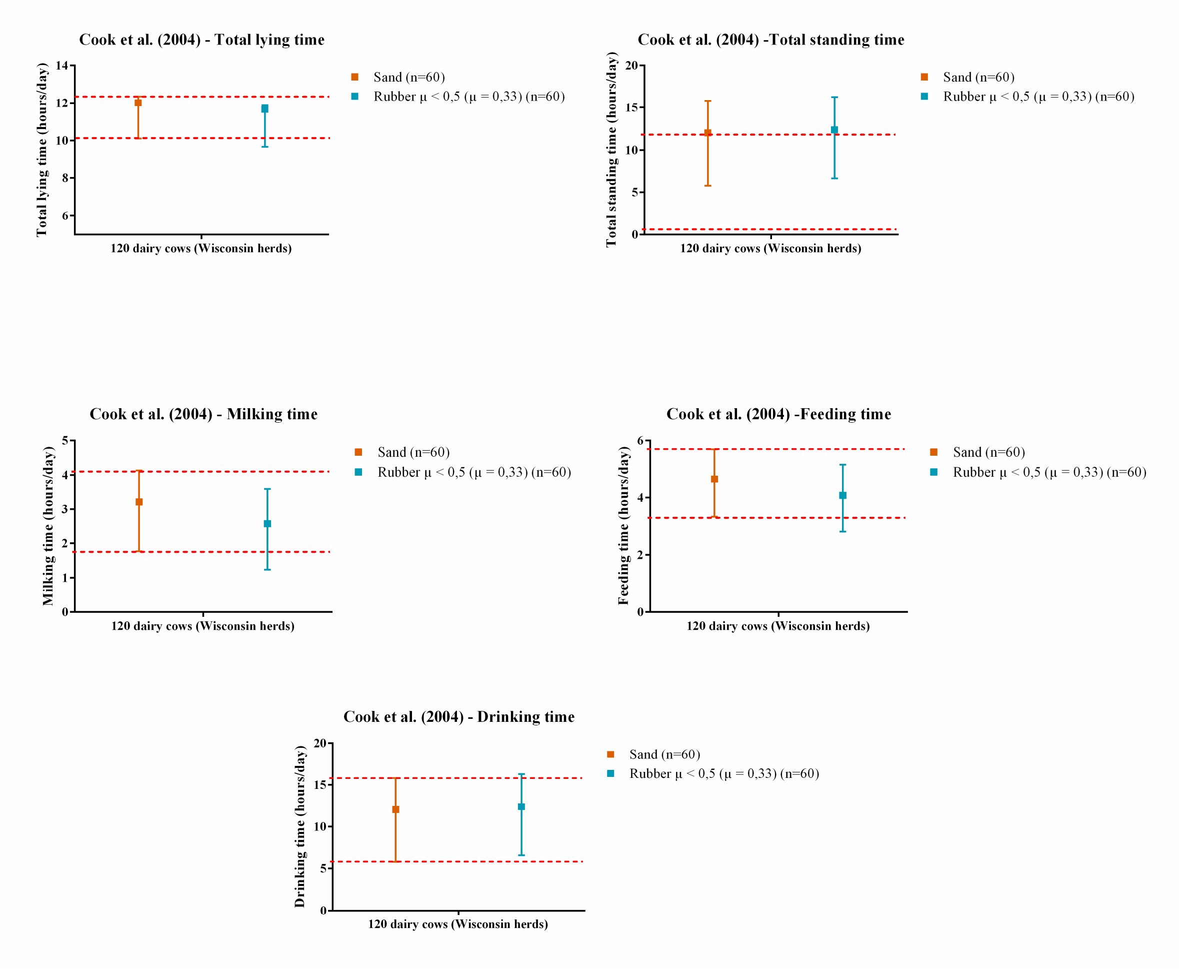

Figure S6. Behavior parameters for sand and rubber surfaces.

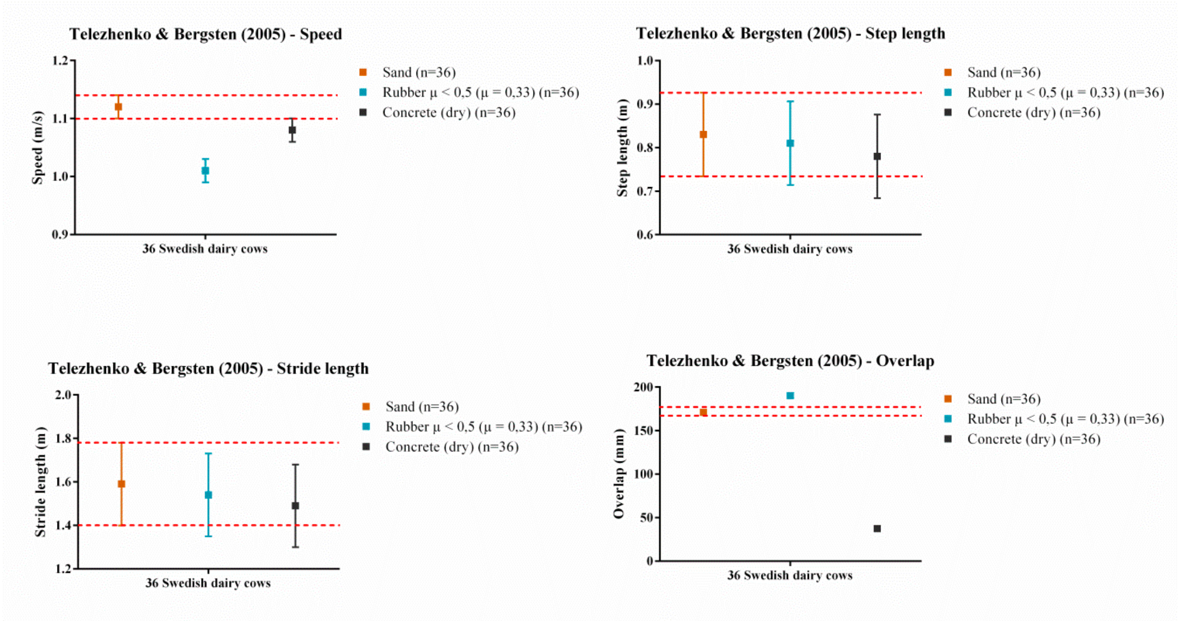

Figure S7. Kinematics parameters for sand, rubber and concrete surfaces.

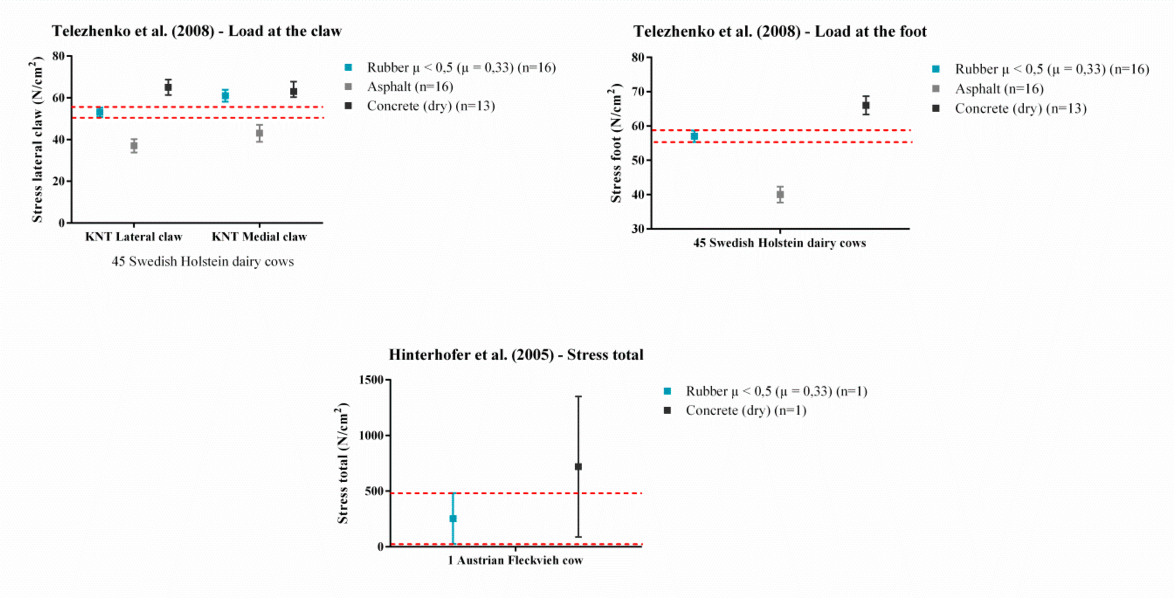

Figure S8. Kinetics parameters for rubber, asphalt and concrete surfaces.

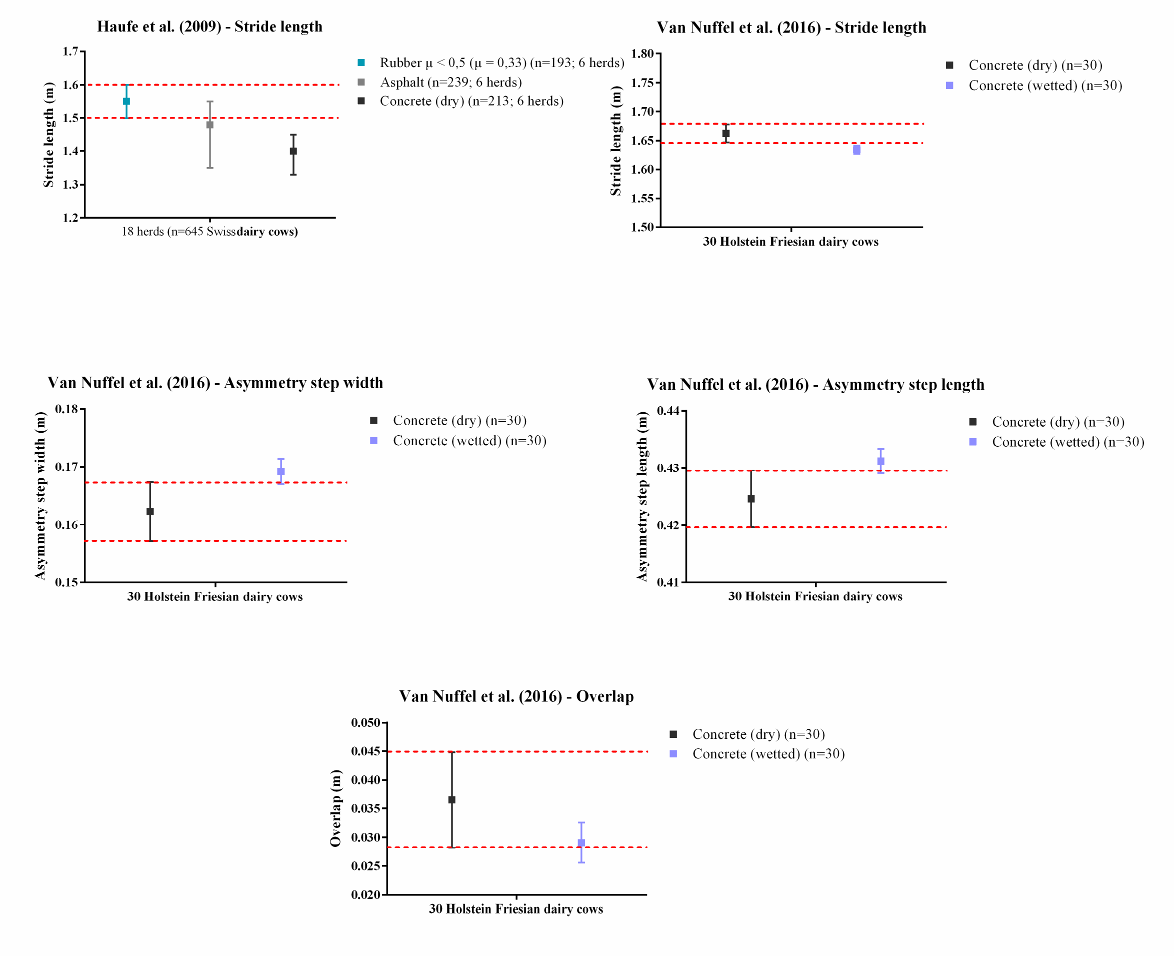

Figure S9. Kinematics parameters for rubber, asphalt and concrete (soak factor) surfaces.

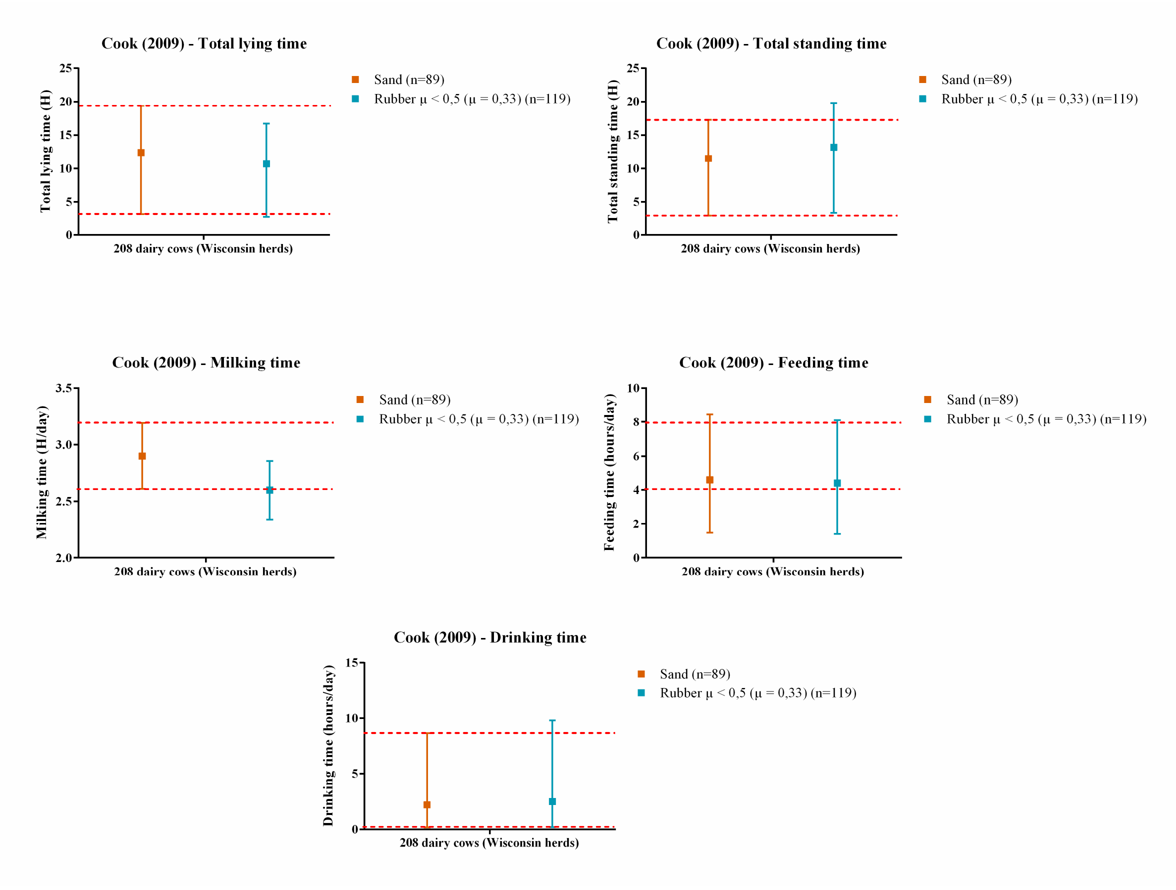

Figure S10. Behavior parameters for sand and rubber surfaces.

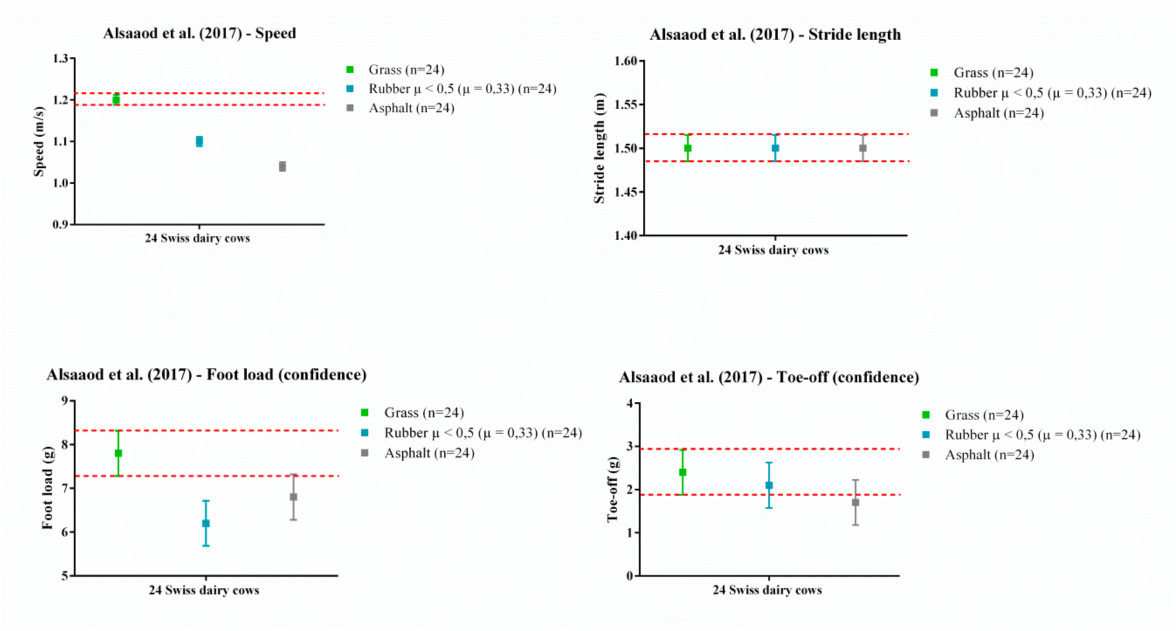

Figure S11. Kinematics and Kinetics parameters for grass, rubber and asphalt surfaces.

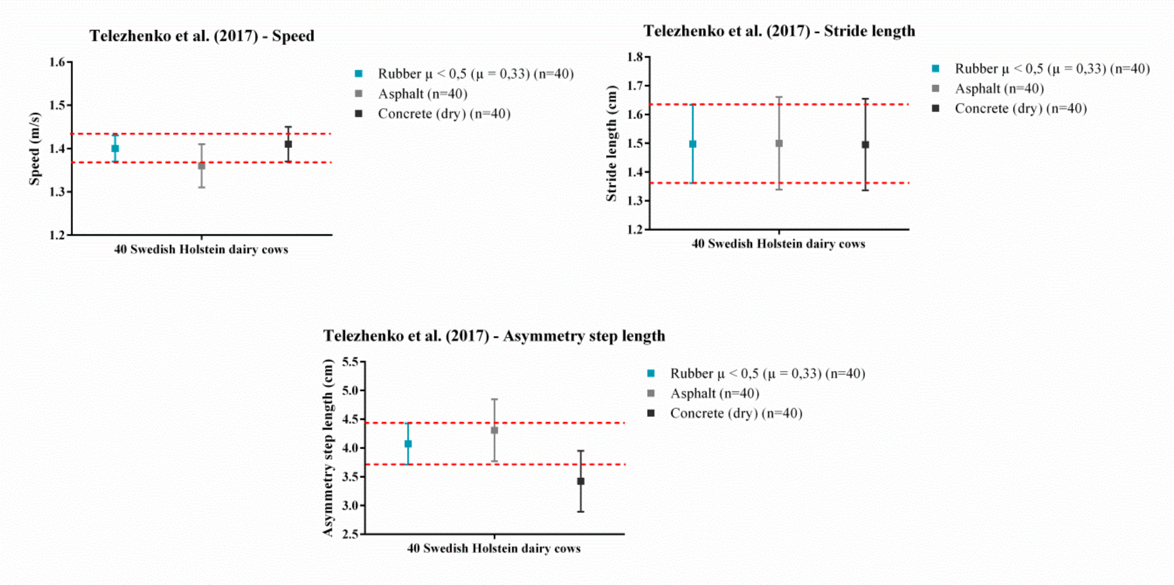

Figure S12. Kinematics parameters for rubber, asphalt and concrete surfaces.

References

8. Herlin, A.H.; Drevemo, S. Investigating locomotion of dairy cows by use of high speed cinematography. *Equine Vet J Suppl.* **1997**;(23):106–9. https://doi.org/10.1111/j.2042-3306.1997.tb05066.x
9. Phillips, C.J.; Morris, I.D. The locomotion of dairy cows on concrete floors that are dry, wet, or covered with a slurry of excreta. *J Dairy Sci.* **2000**;*83*(8):1767–72. https://doi.org/10.3168/jds.S0022-0302(00)75047-8
10. Phillips, C.J.; Morris, I.D. The locomotion of dairy cows on floor surfaces with different frictional properties. *J Dairy Sci.* **2001**;*84*(3):623–8. https://doi.org/10.3168/jds.S0022-0302(01)74517-1
13. Van Nuffel, A.; Van De Gucht, T.; Saeys, W.; Sonck, B.; Opsomer, G.; Vangeyte, J.; et al. Environmental and cow-related factors affect cow locomotion and can cause misclassification in lameness detection systems. *Animal*. **2016**;*10*(9):1533–41. https://doi.org/10.1017/s175173111500244x
14. Alsaaod, M.; Huber, S.; Beer, G.; Kohler, P.; Schüpbach-Regula, G.; Steiner, A. Locomotion characteristics of dairy cows walking on pasture and the effect of artificial flooring systems on locomotion comfort. *J Dairy Sci*. **2017**;*100*(10):8330–7. https://doi.org/10.3168/jds.2017-12760
16. Hinterhofer, C.; Ferguson, J.C.; Apprich, V.; Haider, H.; Stanek, C. A finite element model of the bovine claw under static load for evaluation of different flooring conditions. *N Z Vet J*. **2005**;*53*(3):165–70. https://doi.org/10.1080/00480169.2005.36501
22. Solano, L.; Barkema, H.W.; Pajor, E.A.; Mason, S.; LeBlanc, S.J.; Zaffino Heyerhoff, J.C.; et al. Prevalence of lameness and associated risk factors in Canadian Holstein-Friesian cows housed in freestall barns. *J Dairy Sci*. **2015**;*98*(10):6978–91. https://doi.org/10.3168/jds.2015-9652
27. Cook, N.B.; Bennett, T.B.; Nordlund, K.V. Effect of free stall surface on daily activity patterns in dairy cows with relevance to lameness prevalence. *J Dairy Sci*. **2004**;*87*(9):2912–22. https://doi.org/10.3168/jds.S0022-0302(04)73422-0
29. Frankena, K.; Somers, J.G.C.J.; Schouten, W.G.P.; van Stek, J.V.; Metz, J.H.M.; Stassen, E.N.; et al. The effect of digital lesions and floor type on locomotion score in Dutch dairy cows. *Prev Vet Med.* **2009**;*88*(2):150–7. https://doi.org/10.1016/j.prevetmed.2008.08.004
31. Fabian, J.; Laven, R.A.; Whay, H.R. The prevalence of lameness on New Zealand dairy farms: a comparison of farmer estimate and locomotion scoring. *Vet J*. **2014**;*201*(1):31–8. https://doi.org/10.1016/j.tvjl.2014.05.011
33. Husfeldt, A.W.; Endres, M.I. Association between stall surface and some animal welfare measurements in freestall dairy herds using recycled manure solids for bedding. *J Dairy Sci*. **2012**;*95*(10):5626–34. https://doi.org/10.3168/jds.2011-5075
34. Telezhenko, E.; Bergsten, C. Influence of floor type on the locomotion of dairy cows. *Appl. Anim. Behav. Sci*. **2005**, *93*, 183–97. https://doi.org/10.1016/j.applanim.2004.11.021
35. Rahman, M.A.; Imtiaz, M.A.; Ahaduzzaman, M.; Ghosh, K.K.; Masud, A.A.; Chowdhury, S.; et al. Effects of flooring and rearing system on hoof health of dairy cows in some selected areas of Bangladesh. *Anim sci j Pak*. **2014**;*43*(2):132–7. https://doi.org/10.3329/bjas.v43i2.20714
36. Salfer, J.A.; Siewert, J.M.; Endres, M.I. Housing, management characteristics, and factors associated with lameness, hock lesion, and hygiene of lactating dairy cattle on Upper Midwest United States dairy farms using automatic milking systems. *J Dairy Sci*. **2018**;*101*(9):8586–94. https://doi.org/10.3168/jds.2017-13925
58. Faye, B.; Lescourret, F. Environmental factors associated with lameness in dairy cattle. *Prev Vet Med*. **1989**;*7*(4):267–87. https://doi.org/10.1016/0167-5877(89)90011-1

59. Frankena, K.; van Keulen, K.A.S.; Noordhuizen, J.P.; Noordhuizen-Straasen, E.N.; Gundelach, J.; de Jong, D.-J.; et al. A cross-sectional study into prevalence and risk indicators of digital haemorrhages in female dairy calves. *Prev Vet Med*. **1992**;*14*(1–2):1–12. https://doi.org/10.1016/0167-5877(92)90079-U
61. Cook, N.B. *Free-stall design for maximum cow comfort* [Internet]. Haasnutrition.com. http://haasnutrition.com/wp-content/uploads/2015/09/FreeStallDesign-cook.pdf.
Accessed 9 March 2021.
68. Cook, N.B. Prevalence of lameness among dairy cattle in Wisconsin as a function of housing type and stall surface. *J Am Vet Med Assoc*. **2003**;*223*(9):1324–8. https://doi.org/10.2460/javma.2003.223.1324
69. Vokey, F.J.; Guard, C.L.; Erb, H.N.; Galton, D.M. Effects of alley and stall surfaces on indices of claw and leg health in dairy cattle housed in a free-stall barn. *J Dairy Sci*. **2001**;*84*(12):2686–99. https://doi.org/10.3168/jds.s0022-0302(01)74723-6
70. Espejo, L.A.; Endres, M.I.; Salfer, J.A. Prevalence of lameness in high-producing holstein cows housed in freestall barns in Minnesota. *J Dairy Sci*. **2006**;*89*(8):3052–8. https://doi.org/10.3168/jds.s0022-0302(06)72579-6
71. Haufe, H.C.; Gygax, L.; Steiner, B.; Friedli, K.; Stauffacher, M.; Wechsler, B. Influence of floor type in the walking area of cubicle housing systems on the behaviour of dairy cows. *Appl Anim Behav Sci*. **2009**;*116*(1):21–7. https://doi.org/10.1016/j.applanim.2008.07.004
72. Telezhenko, E.; Magnusson, M.; Bergsten, C. Gait of dairy cows on floors with different slipperiness. *J Dairy Sci*. **2017**;*100*(8):6494–503. https://doi.org/10.3168/jds.2016-12208
73. Telezhenko, E.; Bergsten, C.; Magnusson, M.; Ventorp, M.; Nilsson, C. Effect of different flooring systems on weight and pressure distribution on claws of dairy cows. *J Dairy Sci*. **2008**;*91*(5):1874–84. https://doi.org/10.3168/jds.2007-0742
74. Cramer, G. Quantification of foot lesions and an evaluation of early detection methods for lameness in Ontario dairy farms. **2007**. https://atrium.lib.uoguelph.ca/xmlui/handle/10214/20472. Accessed 9 Mar 2021.
75. Cook, N.B.; Hess, J.P.; Foy, M.R.; Bennett, T.B.; Brotzman, R.L. Management characteristics, lameness, and body injuries of dairy cattle housed in high-performance dairy herds in Wisconsin. *J Dairy Sci*. **2016**;*99*(7):5879–91. https://doi.org/10.3168/jds.2016-10956
77. Haley, D.B.; de Passillé, A.M.; Rushen, J. Assessing cow comfort: effects of two floor types and two tie stall designs on the behaviour of lactating dairy cows. *Appl Anim Behav Sci*. **2001**;*71*(2):105–17. 10.1016/s0168-1591(00)00175-1
